# Imaging evaluation of bone tumors

**DOI:** 10.1590/0100-3984.2016.49.3e2

**Published:** 2016

**Authors:** André Yui Aihara

**Affiliations:** 1 PhD, Collaborator in the Department of Diagnostic Imaging at the Escola Paulista de Medicina - Universidade Federal de São Paulo (EPM-Unifesp), Radiologist at DASA and AACD-SP, São Paulo, SP, Brazil. E-mail: andre.yui.aihara@gmail.com.

The radiographic approach to bone tumors consists in analyzing a certain lesion in an organized manner, paying attention to specific radiographic features such as location, margins and transition zones, periosteal reaction patterns, mineralization, lesion size, and whether or not soft tissue components are present^(1,2)^.

Patient age and determining whether the lesion(s) is single or multiple are fundamental clinical data for diagnosis. Some types of tumors have a predilection for certain age groups^(2)^. With the exception of multiple myeloma, primary malignant bone tumors are typically solitary lesions, whereas benign tumors tend to present as multiple lesions^(1,2)^.

This type of semiological approach has neither the objective nor the capacity to arrive at a final histological diagnosis. However, it narrows the range of differential diagnoses and indicates the most appropriate course of action from that point onward^(2,3)^.

With this semiological approach to conventional radiography, it is possible to reduce the number of differential diagnoses, often eliminating the need for more advanced imaging methods such as computed tomography (CT) or magnetic resonance imaging (MRI). Typical examples of such diagnoses are simple bone cyst, fibrous cortical defect, and non-ossifying fibroma^(3)^, which normally preclude the need for evaluation methods other than conventional radiography.

If necessary, CT can be helpful in determining the pattern of calcification in the matrix of the lesion, in identifying occult bone destruction, or even in localizing the nidus of an osteoid osteoma^(1,2)^. It can also serve to guide the collection of tissue for histological analysis^(4)^ and less invasive treatments of certain tumors, such as osteoid osteoma^(5)^.

MRI has become the standard imaging examination for determining the local extent of a tumor and is frequently capable of providing a better characterization of the components of the lesions, such as cystic areas and adipose, fibrous, or chondral tissue^(2)^. However, it is a costly method that is not always necessary, as explained above. Advanced MRI techniques, such as diffusion, spectroscopy, dynamic tissue perfusion, and in-phase/out-of-phase imaging, show promise for the evaluation of tumors. Together with conventional MRI, these advanced techniques increase diagnostic accuracy and improve evaluation of treatment responses^(6)^.

Positron emission tomography combined with multidetector computed tomography has proven to be a valuable imaging tool for staging, restaging and the evaluation of treatment response in patients with tumors, due to its capacity to provide additional physiological information^(7)^.

In this issue of **Radiologia Brasileira**, Andrade Neto et al.^(8)^ present a pictorial essay in which they review the conventional radiography aspects of the main knee bone tumors seen in clinical practice. In a quite didactic manner, the essay divides expansile knee lesions into pseudotumors, bone-forming tumors, cartilage-forming tumors, bone marrow tumors, and other connective tissue tumors. It also makes a concise review of each of the tumor types discussed, providing an excellent opportunity to learn or recall concepts.
